# Impact of deep learning image reconstruction on CT radiomics reproducibility in patients with pulmonary nodules

**DOI:** 10.1186/s13244-026-02417-y

**Published:** 2026-10-13

**Authors:** Huiyuan Zhu, Tingle Wei, Luna Wang, Lin Zhu, Qunhui Chen, Guangyu Tao, Linlin Sun, Xiwen Sun, Zike Huang, Hong Yu

**Affiliations:** 1 Department of Radiology, Shanghai Chest Hospital, Shanghai Jiao Tong University School of Medicine, Shanghai, China; 2 Department of Radiology, Shanghai Pulmonary Hospital, School of Medicine, Tongji University, Shanghai, China; 3 Department of Radiation Oncology, Fudan University Shanghai Cancer Center, Shanghai, China; 4 Department of Oncology, Shanghai Medical College, Fudan University, Shanghai, China

**Keywords:** Radiomics, Deep learning, Image reconstruction, Computed tomography, Pulmonary nodule

## Abstract

**Objective:**

Ensuring CT feature reproducibility across imaging protocols and reconstruction algorithms is essential for the generalizability of radiomics applications. This study aimed to evaluate the reproducibility of CT radiomic features in pulmonary nodules using deep learning image reconstruction (DLIR) compared with conventional reconstruction techniques under different radiation doses.

**Materials and methods:**

We included 94 patients between March and June 2023. Standard- and low-dose CT images were scanned to assess the impact of dose reduction on feature reproducibility. DLIR strengths at low (DLIR-L), medium (DLIR-M), and high (DLIR-H), representing progressive levels of noise suppression, were compared against filtered back projection (FBP) and adaptive statistical iterative reconstruction-V at 50% strength (ASIR-V-50%) reconstructions. 2264 features were extracted from pulmonary nodules. The concordance correlation coefficient (CCC) and the coefficient of variation (CV) were used to evaluate inter-dose reproducibility, inter-reconstruction reproducibility, and feature variability.

**Results:**

The mean CCC for inter-dose reproducibility using DLIR-H, ASIR-V-50%, and FBP was 0.934 ± 0.104, 0.922 ± 0.128, and 0.922 ± 0.113, respectively (*p* = 0.055), with 29.8%, 27.9%, and 20.2% of features showing near-perfect agreement. In low-dose CT, DLIR-L achieved the highest inter-reconstruction reproducibility against ASIR-V-50% (0.950 ± 0.094), with 39.4% of features showing near-perfect agreement. The overall CV across reconstructions was 9.87 ± 20.32%, with 74.0% of features highly stable. Part-solid nodules showed the lowest variability (7.17 ± 13.48%, *p* < 0.001). Reproducibility improved significantly with increasing nodule size (*p* < 0.001).

**Conclusion:**

DLIR improves the radiomic feature reproducibility in pulmonary nodules, making it particularly suitable for low-dose CT protocols. However, the robustness tends to decrease with higher DLIR strengths. Nodule characteristics significantly influence variability.

**Key Points:**

***Question*** The impact of DLIR on radiomic feature reproducibility across radiation doses and reconstruction methods in pulmonary nodules remains unknown.

***Findings*** DLIR-H demonstrated high inter-dose reproducibility, while DLIR-L achieved the highest inter-reconstruction reproducibility; feature variability decreased significantly with increasing nodule size, with 21 robust features identified.

***Critical relevance statement***This study critically assesses reconstruction-related instability in CT radiomics and identifies DLIR settings and nodule characteristics that influence feature robustness, supporting protocol optimization and more reliable quantitative pulmonary nodule assessment in clinical radiology.

**Graphical Abstract:**

**Figure:**
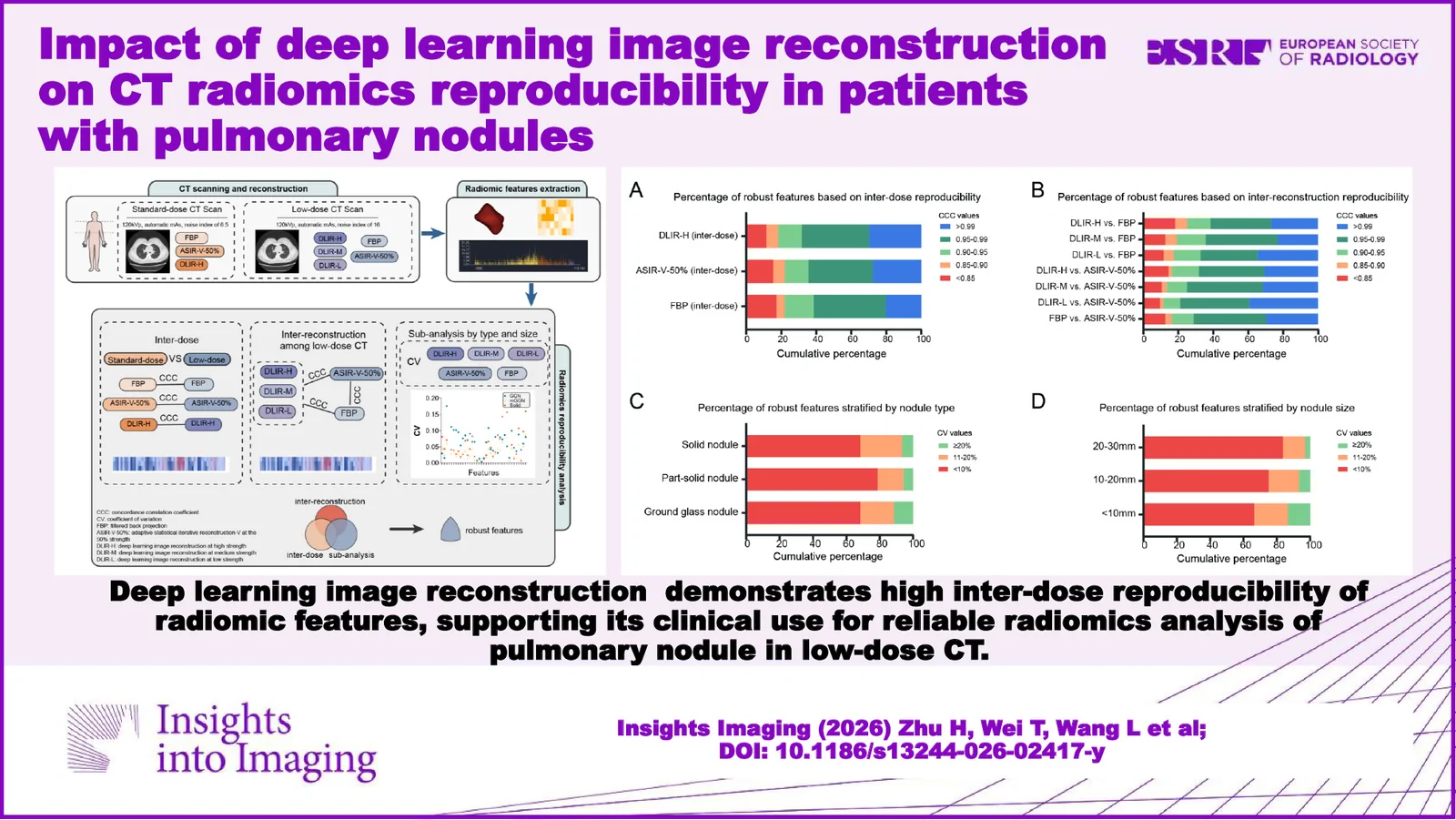

## Introduction

Radiomics plays an important role in clinical research by extracting high-dimensional features, thereby converting medical images into quantitative mathematical data [1]. Recent studies have demonstrated that radiomics is a powerful tool for predicting tumor invasiveness [2], identifying pathological subtypes [3] and genetic mutations [4], and stratifying survival risk [5]. However, despite these promising results, radiomics has not yet achieved a definitive breakthrough into the clinical mainstream. A major factor limiting the generalizability of radiomic features is their lack of robustness, even though substantial efforts have been made to standardize imaging biomarkers [6, 7]. In particular, increasing evidence suggests that dose levels and image reconstruction algorithms are key sources of inconsistency and instability in radiomic features [8–10].

Deep learning image reconstruction (DLIR), a novel algorithm that mimics high-dose and high-quality filtered back projection (FBP) images using a deep learning-based convolutional neural network, has been developed to maintain image quality while reducing radiation dose [11–13]. In pulmonary nodule imaging, DLIR has been demonstrated to enhance image quality and improve lesion visualization [14, 15]. However, as a recently introduced technology, the reproducibility of radiomic features derived from DLIR images has not been fully explored. Some phantom studies have reported that DLIR images exhibit lower reproducibility than those reconstructed using adaptive statistical iterative reconstruction-V (ASIR-V) [16, 17], while other studies suggested that DLIR can improve image quality and yield more reliable radiomic features [18]. Nevertheless, due to the inherent differences between phantom materials and human tissue, phantom studies cannot fully simulate the biological complexity of clinical lesions. As such, their findings may not be directly applicable to patient data. Therefore, further investigation into the variability of radiomic features derived from DLIR and traditional CT reconstructions is warranted.

Therefore, the purpose of this study was to evaluate the reproducibility of radiomic features extracted from pulmonary nodules imaged at different radiation doses (low-dose CT and standard-dose CT) and reconstructed using various algorithms (DLIR, FBP, and ASIR-V), in order to assess the impact of DLIR on feature stability.

## Materials and methods

### Patients

**Fig. 1:**
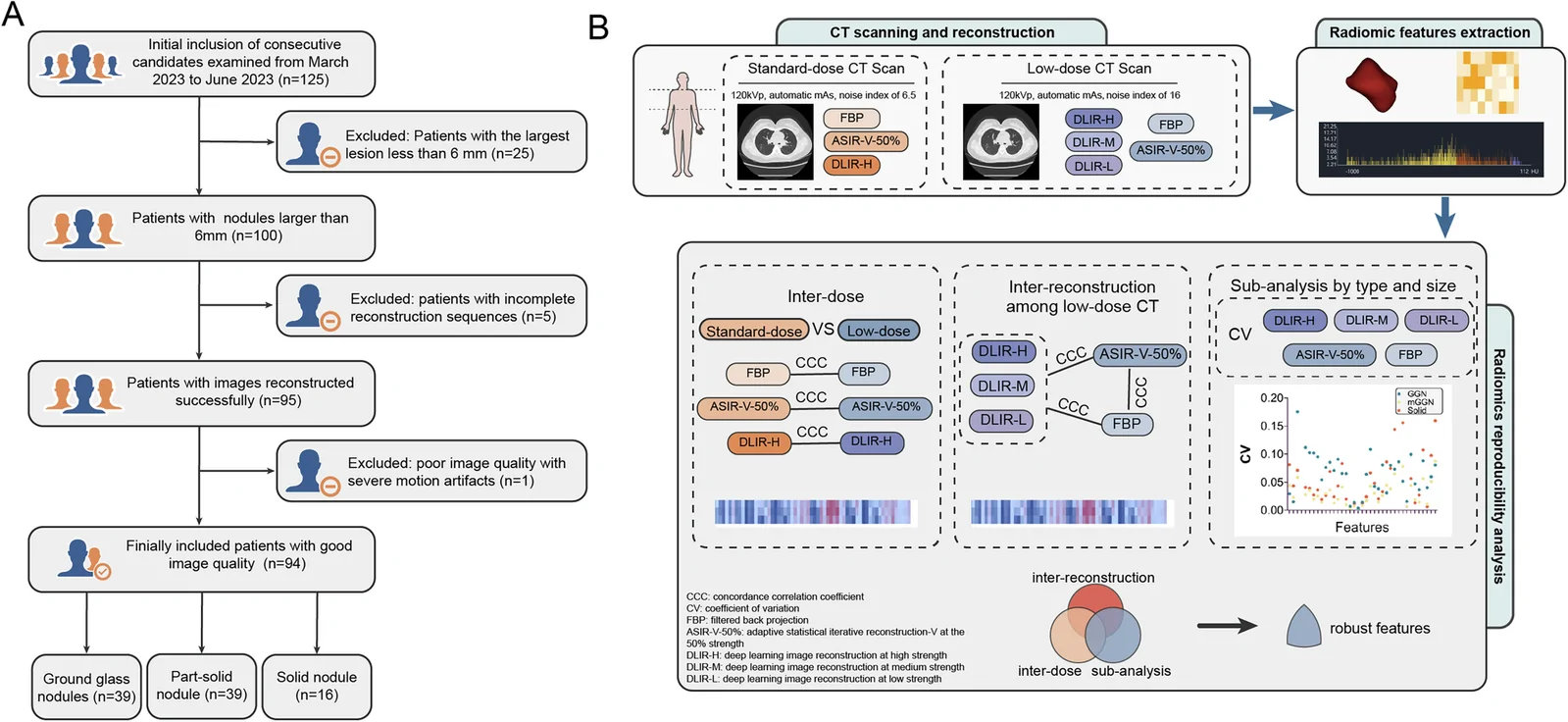
The flowchart for the patient inclusion (**A**) and research pipeline of this study (**B**)

This study was a retrospective second analysis of a previous prospective study. Ethical approval was obtained from the institutional review board of our hospital (ethics approval no. IS23088) and informed consent was waived. A total of 125 consecutive patients with prior chest CT findings of pulmonary nodules admitted between March and June 2023 were initially enrolled based on the following inclusion criteria: (1) completion of both standard-dose and low-dose CT scans; and (2) presence of pulmonary nodules, as detected and confirmed by radiologists. Exclusion criteria were as follows: (1) the largest pulmonary lesion measuring less than 6 mm; (2) incomplete image reconstruction sequences; and (3) poor image quality with severe motion artifacts. Ultimately, 31 patients were excluded, resulting in a final study cohort of 94 patients. For patients with multiple lesions, the largest lesion by maximum overall diameter was selected to ensure an adequate voxel count for radiomics analysis. A detailed flowchart of patient inclusion is presented in Fig. 1A.

### Image acquisition and reconstruction

All CT examinations were conducted using a 256-row CT scanner (Revolution CT, GE HealthCare) without the use of contrast media. Standard-dose scans were performed with a tube voltage of 120 kVp and automatic tube current modulation, with a noise index of 6.5. Subsequently, a low-dose scan was performed using the same tube voltage but with a noise index increased to 16 to reduce tube current. Additional scanning parameters included a 500 ms rotation time, 0.625 × 128 mm collimation, and a pitch of 0.992:1. All scans were obtained during two successive inspiratory breath-holds in the supine position, separated by an interval of 4.9 s. The effective doses for the standard-dose and low-dose scans were 5.43 ± 0.78 mSv and 0.86 ± 0.14 mSv, respectively.

All images were reconstructed on a dedicated workstation using vendor-specific, proprietary algorithms (GE Healthcare). Standard-dose CT images were reconstructed using FBP, ASIR-V at 50% strength (ASIR-V-50%, the routine clinical setting at our institution), and the DLIR algorithm (TrueFidelity) at high strength (DLIR-H). Low-dose CT images were reconstructed using FBP, ASIR-V-50%, DLIR-H, DLIR at medium strength (DLIR-M), and DLIR at low strength (DLIR-L), with the different DLIR strengths corresponding to different levels of noise reduction. All images were reconstructed using a standard kernel with a 1.25 mm thickness and interval. A lung display window with a width of 1500 HU and a level of −400 HU was applied. The workflow of this study is illustrated in Fig. 1B.

**Fig. 2:**
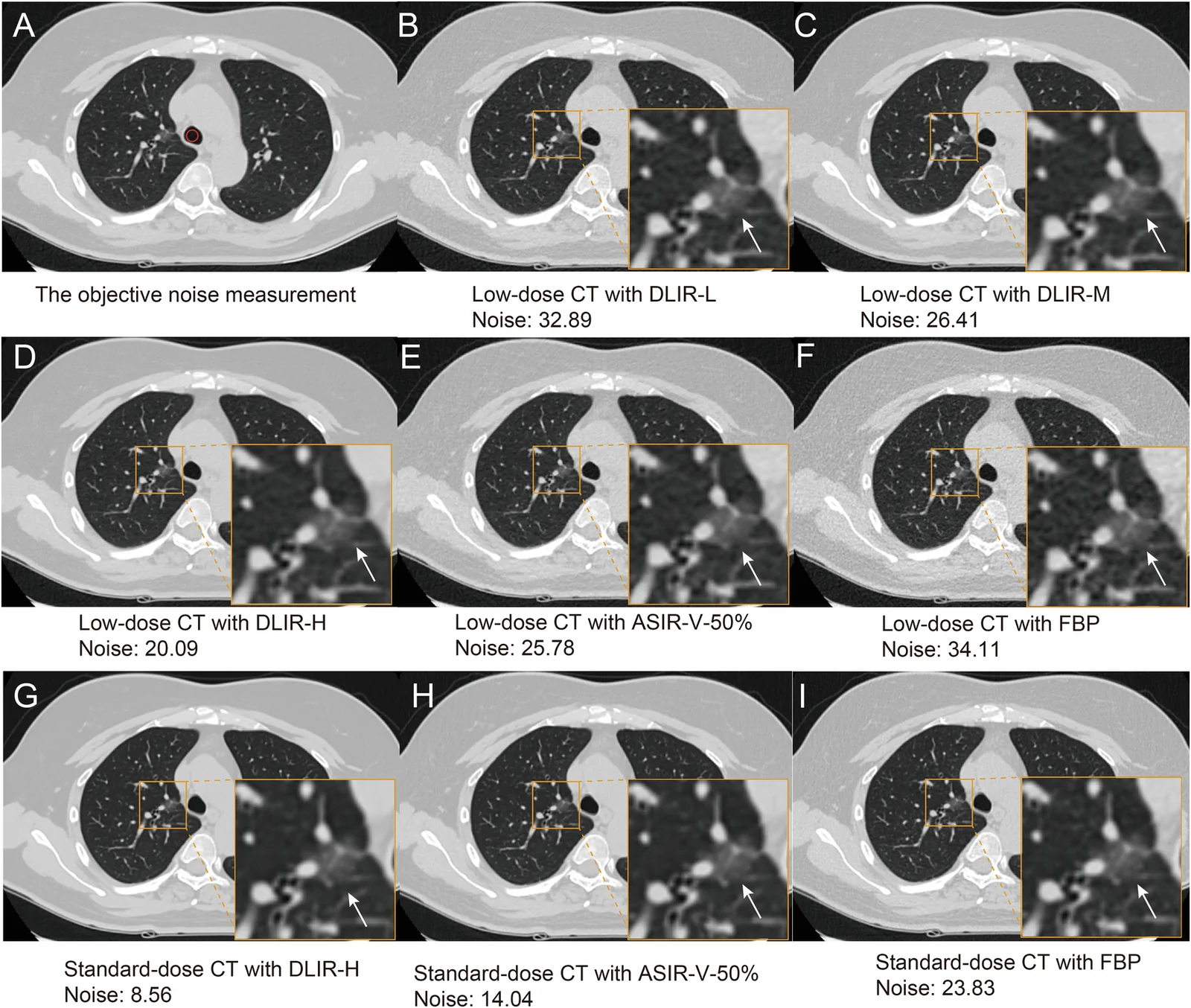
Images of a 70-year-old female patient with a subsolid nodule in the right upper lobe of the lung, pathologically confirmed as invasive adenocarcinoma. Images reconstructed with high-strength deep learning image reconstruction (DLIR-H) show lower image noise compared with adaptive statistical iterative reconstruction V at 50% strength (ASIR-V-50%) and FBP. The objective noise measurement (**A**). A fixed-size circular ROI was placed in the trachea, and objective noise was quantified as the standard deviation of Hounsfield units within the ROI. Low-dose CT images were reconstructed using DLIR at low strength (**B**), DLIR at medium strength (**C**), DLIR-H (**D**), ASIR-V-50% (**E**), and FBP (**F**). Standard-dose CT images were reconstructed using DLIR-H (**G**), ASIR-V-50% (**H**), and FBP (**I**). Boxes with magnified illustrations of the nodules have been added to improve visibility

For each reconstruction series, a fixed-size circular region-of-interest (ROI) with a diameter of 5 mm was placed in the trachea, and objective noise was quantified as the standard deviation of Hounsfield units within the ROI (Figs. 2A and S1A).

### Segmentation and radiomic features extraction

Nodule segmentation was automatically performed independently on each reconstructed image set using the uAI Research Portal (Shanghai United Imaging Intelligence) to generate a 3D ROI [19]. Manual corrections for ROI selection were performed by a radiologist with 2 years of experience in chest imaging and reviewed by another radiologist with 32 years of experience. Both radiologists were blinded to the image acquisition parameters and clinical information.

For each ROI, a total of 2264 features were extracted from both the original and filtered images. A total of 24 different filtered image sets were generated by applying individual filters separately (Supplemental Material). The extracted features were grouped into three categories: 18 first-order features, 14 shape-based features (only extracted from the original images), and 72 texture features (Supplemental material). All the extracted features were in concordance with the standard set by the Imaging Biomarker Standardization Initiative (IBSI) [7].

### Radiomics reproducibility analysis

To assess the impact of dose reduction and reconstruction algorithms on reproducibility of radiomic features, both inter-dose and inter-reconstruction reproducibility were evaluated by concordance correlation coefficient (CCC) [20]. Inter-dose reproducibility referred to the agreement between radiomic features extracted from standard-dose and low-dose CT images reconstructed with the same method (FBP, ASIR-V-50%, or DLIR-H). Inter-reconstruction reproducibility referred to the agreement between features derived from different reconstruction algorithms using the same low-dose CT scan, thereby assessing whether the reconstruction method itself alters feature values. A CCC value of 0.85 or higher was considered to have good reproducibility [21]. To comprehensively characterize reproducibility patterns, the number of features exceeding CCC thresholds of 0.90, 0.95, and 0.99 was quantified [22, 23]. Mean CCC values were also compared across feature categories and filters to evaluate variability related to feature type and preprocessing.

Sub-analysis according to nodule size and type was performed to assess the variability of features derived from low-dose CT reconstructions using the coefficient of variation (CV) [24, 25]. CV values were interpreted as acceptable (< 10%), moderate but still adequate (11–20%), or too high and inadequate (≥ 20%) [26, 27].

We focused our primary analysis on the 104 original features, as they provide the most direct and basic image information. Results for the filtered features are presented in the Supplementary Material.

### Statistical analysis

Data are presented as mean ± standard deviation for continuous variables, and as frequency and percentage for categorical variables. The Friedman test was used to compare the CCCs and CVs of features, with post-hoc pairwise comparisons adjusted for multiple comparisons using the Bonferroni correction. Pearson’s chi-squared test or Fisher’s exact test was used to compare the numbers of robust features among different groups. All statistical analyses were performed using R statistical software version 4.4.2. A two-sided *p* value < 0.05 was considered statistically significant. Power calculations are presented in the Supplementary Material and Table S1.

## Results

### Patient characteristics

In total, 94 patients (mean age, 56.2 ± 11.9 years; range, 23–77 years) with 94 nodules were enrolled in this study, including 28 (29.8%) men and 66 (70.2%) women. The mean nodule size was 14.9 ± 6.9 mm. There were an equal number of pure ground-glass nodules (GGNs, 39/94, 41.5%) and part-solid nodules (39/94, 41.5%) followed by solid nodules (16/94, 17.0%). A total of 84 nodules were surgically resected and histopathologically confirmed, including 4 benign lesions and 80 malignant nodules (2 mucosa-associated lymphoid tissue lymphomas, 16 adenocarcinomas in situ, 18 minimally invasive adenocarcinomas, and 44 invasive adenocarcinomas). Detailed patient characteristics are summarized in Table 1.

**Table 1:** Participant and nodule characteristics

Variable	No. of patients
No. of patients	94
Age	56.2 ± 11.9
Sex	
Male	28 (29.8%)
Femal	66 (70.2%)
Type	
Ground glass nodule	39 (41.5%)
Part-solid nodule	39 (41.5%)
Solid nodule	16 (17.0%)
Size	14.9 ± 6.9
Pathology	
Benign	4 (4.8%)
Malignant	80 (95.3%)

The noise in standard-dose CT were 25.94 ± 6.64 for FBP, 19.10 ± 5.93 for ASIR-V-50%, and 14.07 ± 4.72 for DLIR-H (*p* < 0.001); in low-dose CT, the noise were 41.78 ± 5.58 for FBP, 31.36 ± 4.93 for ASIR-V-50%, 23.97 ± 4.16 for DLIR-H, 30.22 ± 4.60 for DLIR-M, and 35.83 ± 5.13 for DLIR-L (*p* < 0.001) (Figs. 2 and S1).

### Inter-dose reproducibility

**Fig. 3:**
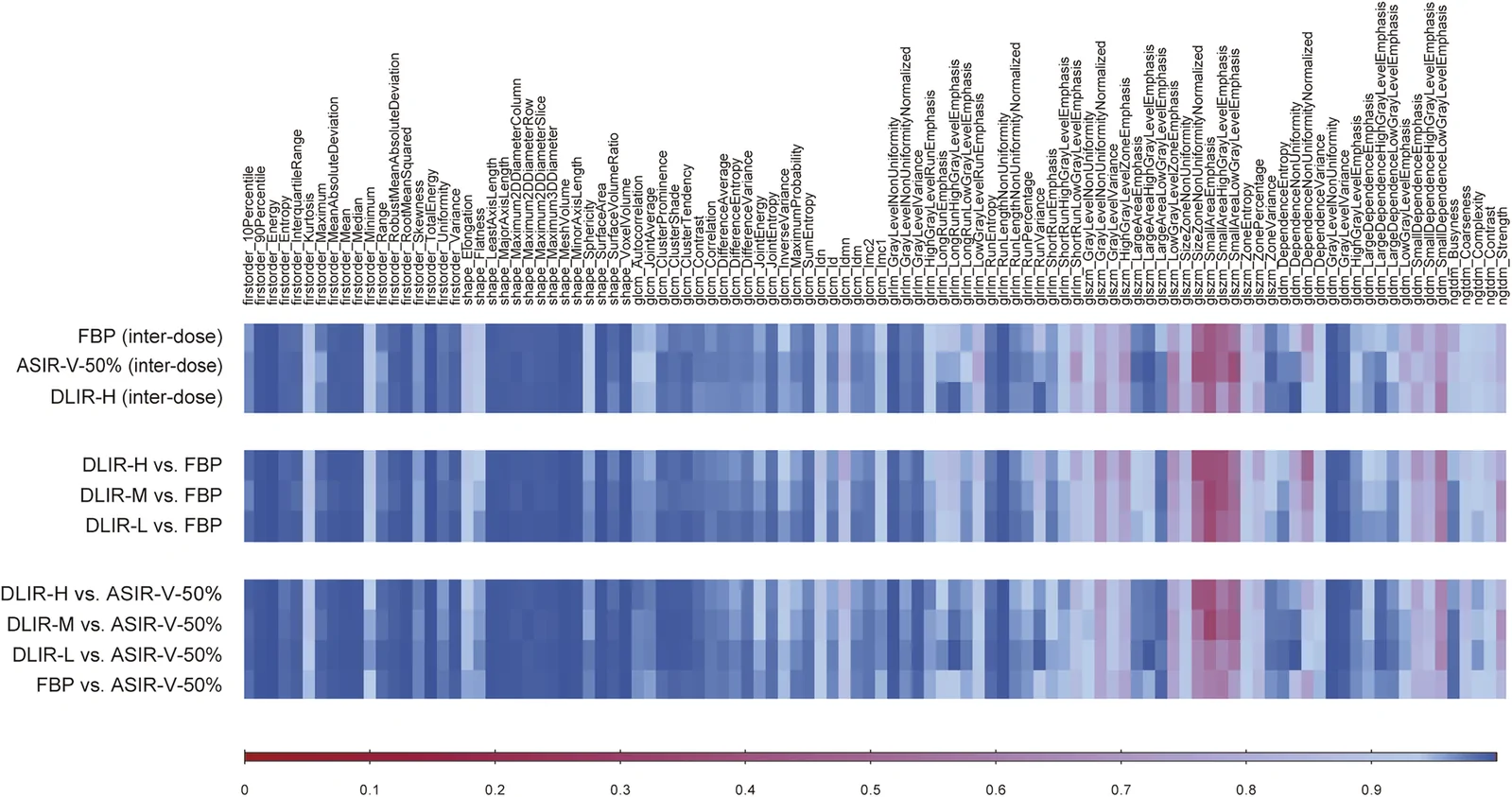
Heat map of the CCC for 104 original radiomic features across different comparisons. CCC values of 0.85 or higher were considered to have good reproducibility

To analyse inter-dose reproducibility, we evaluated the same reconstruction algorithm between standard-dose and low-dose CT images to assess the impact of radiation dose alone. Overall, DLIR-H achieved the highest mean CCC (0.934 ± 0.104), followed by FBP (0.922 ± 0.113) and ASIR-V-50% (0.922 ± 0.128), with borderline statistical significance among the groups (*p* = 0.055). When stratified by feature categories, both the first-order and shape-based features exhibited high reproducibility across all reconstruction methods, with CCC values consistently exceeding 0.977. The texture features showed comparatively lower reproducibility, with DLIR-H (0.914 ± 0.119) significantly outperforming both FBP (0.897 ± 0.127) and ASIR-V-50% (0.896 ± 0.146) (*p* = 0.015). The results are summarized in Table 2 and Fig. 3.

**Table 2:** CCCs of radimoic features between standard-dose and low-dose CT

	FBP	ASIR-V-50%	DLIR-H	*p* value
Mean				
Overall	0.922 ± 0.113	0.922 ± 0.128	0.934 ± 0.104	0.055
First-order features	0.981 ± 0.025	0.981 ± 0.023	0.982 ± 0.023	0.801
Shape-based features	0.977 ± 0.037	0.978 ± 0.034	0.979 ± 0.032	0.807
Texture features	0.897 ± 0.127^*^	0.896 ± 0.146	0.914 ± 0.119	0.015
Number of features				0.820
< 0.85	18 (17.3%)	16 (15.4%)	12 (11.5%)	
≥ 0.85	86 (82.7%)	88 (84.6%)	92 (88.5%)	
≥ 0.90	81 (77.9%)	81 (77.9%)	85 (81.7%)	
≥ 0.95	64 (61.5%)	67 (64.4%)	71 (68.3%)	
≥ 0.99	21 (20.2%)	29 (27.9%)	31 (29.8%)	

*FBP* filtered back projection, *ASIR-V-50%* adaptive statistical iterative reconstruction-V level 50%, *DLIR-H* high-strength deep learning image reconstruction

^*^ *p* < 0.05, compared with CCC of DLIR-H

**Fig. 4:**
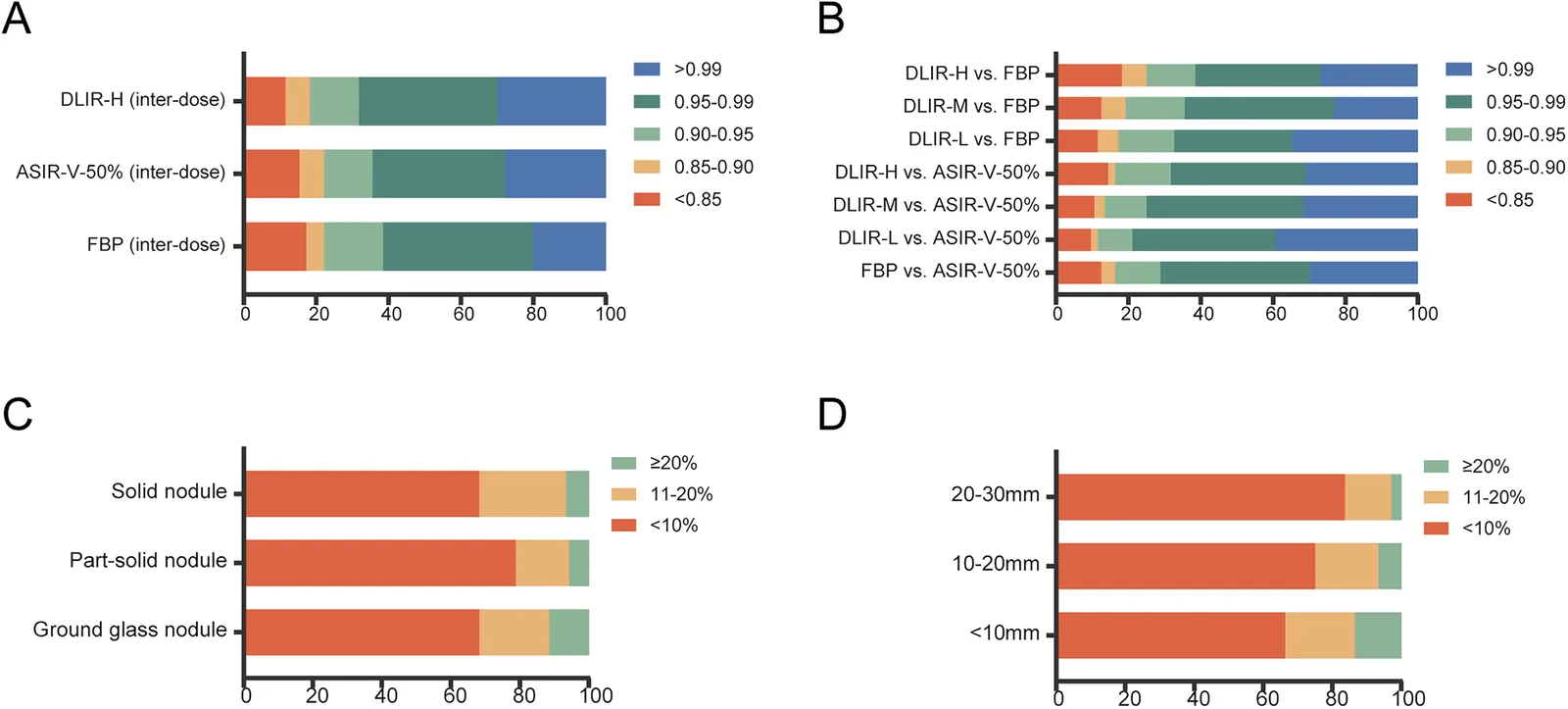
Percentage of robust radiomic features. Histograms show the percentage of robust features based on inter-dose (**A**) and inter-reconstruction reproducibility (**B**), as determined by the CCC. Additionally, histograms illustrate the percentage of robust features stratified by nodule type (**C**) and type size (**D**), as determined by the CV

DLIR-H preserved a higher number of robust features, although the differences were not statistically significant (Table 2 and Fig. 4A). Specifically, 92 (88.5%) features showed CCC ≥ 0.85 with DLIR-H, compared to 88 (84.6%) features with ASIR-V-50% and 86 (82.7%) features with FBP. Similarly, DLIR-H (31/104, 29.8%) maintained the highest number of features with CCC ≥ 0.99, followed by ASIR-V-50% (29/104, 27.9%) and FBP (21/104, 20.2%), suggesting superior stability in feature quantification under low-dose conditions.

CCC values for the 24 filtered image sets are presented in Table S2. The highest CCC values were observed with the Speckle Noise filter (CCC range: 0.930–0.935). In contrast, CCCs of the Wavelet were consistently below 0.745. This indicates that certain transformations may introduce significant variability and should be applied cautiously in radiomics pipelines. Notably, DLIR-H showed non-inferiority compared to FBP or ASIR-V-50% across other filters including box mean, additive gaussian noise, curvature flow, box-sigma, laplacian sharpening, discrete Gaussian, and wavelet.

### Inter-reconstruction reproducibility

To analyse inter-dose reproducibility, we evaluated the CCC between DLIR (L, M, H) and ASIR-V-50%, a commonly used algorithm in low-dose CT noise reduction. For additional context, we also calculated the CCC between FBP and ASIR-V-50%, as well as FBP and the DLIR protocols, providing insight into how DLIR deviates from traditional FBP at low radiation doses. The CCCs of radiomic features derived from low-dose CT images reconstructed using different algorithms are summarized in Table 3 and Fig. 3. Overall, high reproducibility was observed across all reconstruction pairings. Reproducibility between DLIR and ASIR-V-50% (CCC range: 0.933–0.950) was generally superior to that between DLIR and FBP (CCC range: 0.909–0.923), indicating greater consistency between DLIR and ASIR-V-50% reconstructions. However, an inverse relationship was observed between DLIR strength and reproducibility, with increasing DLIR strength leading to reduced CCCs, likely attributable to enhanced noise suppression capabilities. Specifically, the CCC between DLIR-L and ASIR-V-50% was 0.950 ± 0.094, exceeding that between FBP and ASIR-V-50% (0.943 ± 0.087, *p* < 0.001). However, DLIR-H showed lower reproducibility in its comparisons. DLIR-H vs FBP yielded a CCC of 0.909 ± 0.138, and DLIR-H vs ASIR-V-50% yielded a CCC of 0.933 ± 0.116, both significantly lower than the CCC between FBP and ASIR-V-50% (0.943 ± 0.087, both *p* < 0.001). Across all reconstruction comparisons, the first-order and shape-based features maintained excellent reproducibility, with mean CCCs consistently exceeding 0.981. Texture features, however, exhibited lower reproducibility, with CCC values ranging from 0.876 to 0.931.

**Table 3:** CCCs of radimoic features among low-dose CT reconstructed with different algorithm

	FBP vs ASIR-V-50%	DLIR vs ASIR-V-50%			DLIR vs FBP			*p* value
		DLIR-H	DLIR-M	DLIR-L	DLIR-H	DLIR-M	DLIR-L	
Mean								
Overall	0.943 ± 0.087	0.933 ± 0.116^*^	0.938 ± 0.116	0.950 ± 0.094^*^	0.909 ± 0.138^*^	0.923 ± 0.126^*^	0.932 ± 0.118	< 0.001
First-order features	0.989 ± 0.014	0.986 ± 0.020	0.985 ± 0.023	0.990 ± 0.015	0.983 ± 0.028	0.981 ± 0.026^*^	0.986 ± 0.023	< 0.001
Shape-based features	0.991 ± 0.012	0.987 ± 0.020	0.989 ± 0.015	0.992 ± 0.012	0.984 ± 0.026^*^	0.987 ± 0.019	0.990 ± 0.017	< 0.001
Texture features	0.922 ± 0.097	0.909 ± 0.132	0.916 ± 0.134	0.931 ± 0.107^*^	0.876 ± 0.155^*^	0.896 ± 0.143^*^	0.908 ± 0.135	< 0.001
Number of features								0.614
< 0.85	13 (12.5%)	15 (14.4%)	11 (10.6%)	10 (9.6%)	19 (18.3%)	13 (2.5%)	12 (11.5%)	
≥ 0.85	91 (87.5%)	89 (85.6%)	93 (89.4%)	94 (90.4%)	85 (81.7%)	91 (87.5%)	92 (88.5%)	
≥ 0.90	87 (83.7%)	87 (83.7%)	90 (86.5%)	92 (88.5%)	78 (75.0%)	84 (80.8%)	86 (82.7%)	
≥ 0.95	74 (71.2%)	71 (68.3%)	78 (75.0%)	82 (78.8%)	64 (61.5%)	67 (64.4%)	70 (67.3%)	
≥ 0.99	31 (29.8%)	32 (30.8%)	33 (31.7%)	41 (39.4%)	28 (26.9%)	24 (23.1%)	36 (34.6%)	

*FBP* filtered back projection, *ASIR-V-50%* adaptive statistical iterative reconstruction-V level 50%, *DLIR* deep learning image reconstruction, *DLIR-H* high-strength deep learning image reconstruction, *DLIR-M* medium-strength deep learning image reconstruction, *DLIR-L* low-strength deep learning image reconstruction

^*^ *p* < 0.05, compared with CCC of FBP vs ASIR-V-50%

The number of robust features also decreased with increasing DLIR strength (Table 3 and Fig. 4B). The DLIR-L vs ASIR-V-50% pairing retained the highest number of robust features (94/104, 90.4%), slightly surpassing that of FBP vs ASIR-V-50% (91/104, 87.5%). Notably, DLIR-L also preserved the largest proportion of near-perfectly reproducible features, with 41 out of 104 features (39.4%) achieving CCC ≥ 0.99, compared to 23.1–34.6% in other reconstruction comparisons.

Analysis of the filtered image sets (Table S3) revealed that most filters yielded good reproducibility, with mean CCCs generally exceeding 0.868 across all reconstructions. The Speckle Noise achieved the highest reproducibility, with CCCs exceeding 0.923 in all comparisons. However, Wavelet (CCC range: 0.711–0.830), Normalize (CCC range: 0.833–0.910), and LoG (CCC range: 0.832–0.900) exhibited reduced reproducibility. These findings suggest that such transformations may introduce greater variability and may be less suitable for radiomics analyses of low-dose CT imaging.

### Sub-analysis of nodule type and nodule size

**Fig. 5:**
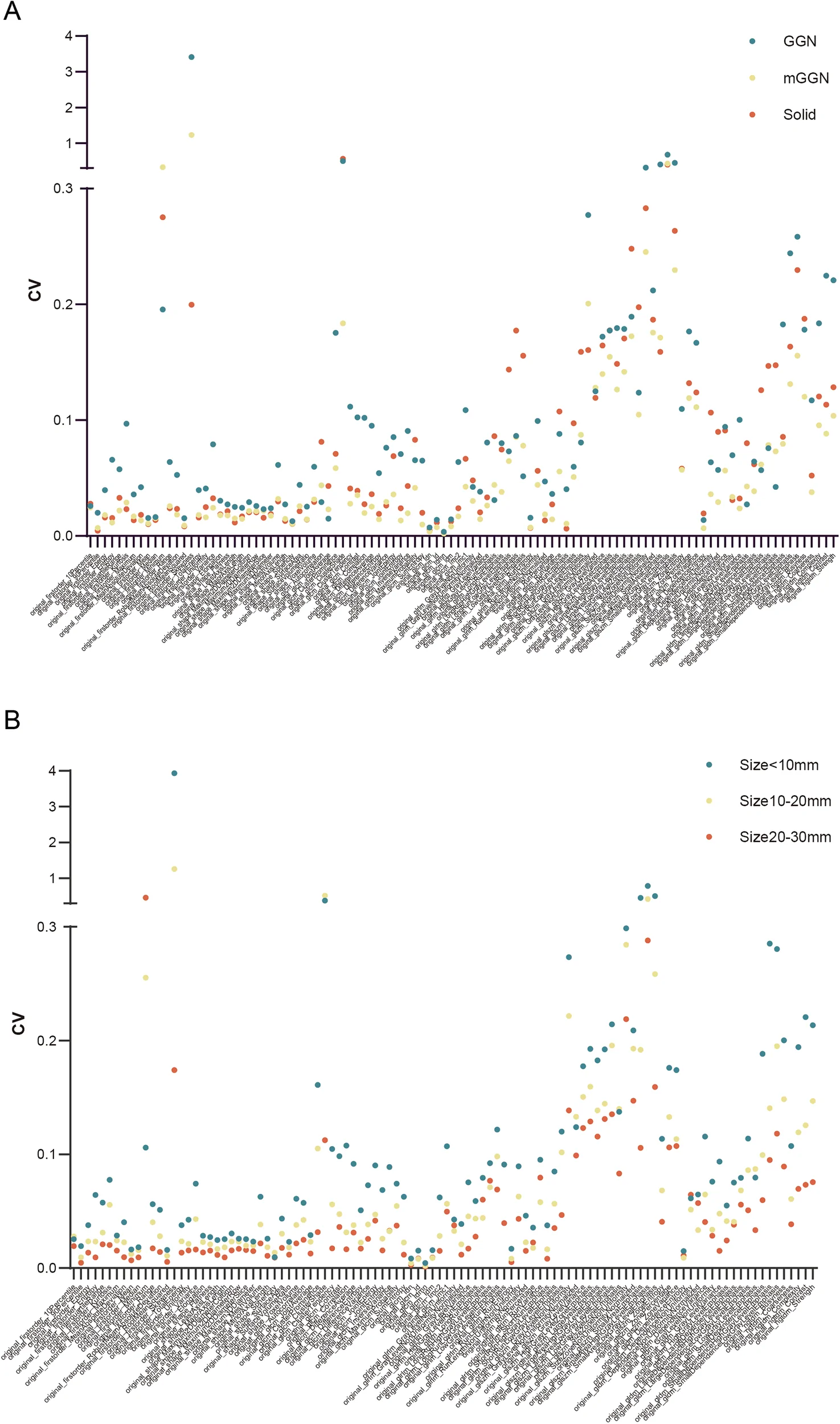
Scatter plot of the CV for radiomic features. **A** Distribution of CV values for each radiomic feature stratified by nodule type, including pure ground-glass nodules, part-solid nodules, and solid nodules. **B** Distribution of CV values stratified by nodule size: < 10 mm, 10–20 mm, and 20–30 mm. Each dot represents the CV of an individual feature, with color indicating the corresponding subgroup. CV values were interpreted as acceptable (< 10%), moderate but still adequate (11–20%), or too high and inadequate (≥ 20%)

Radiomic feature reproducibility varied by nodule type (Table 4 and Fig. 5A). Part-solid nodules showed significantly lower variability, with a mean CV of 7.17 ± 13.48%, compared to solid nodules (8.24 ± 9.06%, *P* < 0.001) and GGNs (13.24 ± 34.21%, *p* < 0.001). The proportion of features with CV < 10% was highest in part-solid nodules (82/104, 78.8%), compared to solid nodules (71/104, 68.3%) and GGNs (71/104, 68.3%) (Fig. 4C). Additionally, GGNs exhibited the greatest variability, with the highest number of features showing CV ≥ 20% (12/104, 11.5%), suggesting that radiomic quantification in GGNs may be more sensitive to reconstruction-related variability.

**Table 4:** CVs of 104 original radimoic features

	CV		Number of features			
	Mean	*p* value	CV < 10%	10%–20%	CV ≥ 20%	*p* value
Overal	9.87 ± 20.32%		77 (74.0%)	18 (17.3%)	9 (8.7%)	
Type^*^		< 0.001				0.204
Ground-glass nodule	13.24 ± 34.21%		71 (68.3%)	21 (20.2%)	12 (11.5%)	
Part-solid nodule	7.17 ± 13.48%		82 (78.8%)	16 (15.4%)	6 (5.8%)	
Solid nodule	8.24 ± 9.06%		71 (68.3%)	26 (25.0%)	7 (6.7%)	
Size^†^		< 0.001				0.024
< 10 mm	14.18 ± 39.23%		69 (66.3%)	21 (20.2%)	14 (13.5%)	
10–20 mm	8.46 ± 14.30%		78 (75.0%)	19 (18.3%)	7 (6.7%)	
20–30 mm	5.16 ± 6.47%		87 (83.7%)	14 (13.5%)	3 (2.9%)	

*CV* coefficient of variation

^*^ *p* < 0.001 for all pairwise comparisons of mean CV by type

^†^ *p* < 0.001 for all pairwise comparisons of mean CV by size

Furthermore, reproducibility improved with increasing nodule size (Table 4 and Fig. 5B). Nodules measuring 20–30 mm showed the lowest variability (5.16 ± 6.47%), compared to those measuring 10–20 mm (8.46 ± 14.30%, *p* < 0.001) and those smaller than 10 mm (14.18 ± 39.23%, *p* < 0.001). Notably, 87 (83.7%) features in the 20–30 mm group showed acceptable variability, compared to 78 (75.0%) features in the 10–20 mm group and 69 (66.3%) features in the < 10 mm group, indicating that smaller nodules are more susceptible to measurement variability (Fig. 4D).

Table S4 presents a filter-wise analysis of CVs across different nodule types and sizes. Among all filters, Speckle Noise (8.40 ± 6.73%) and Shot Noise (9.40 ± 9.57%) demonstrated acceptable variability, indicating strong reproducibility. In contrast, the Wavelet showed inadequate reproducibility, with a mean CV of 21.24 ± 59.84%, suggesting that it introduces substantial variability and may compromise the robustness of extracted features.

### Robust features

A total of 21 original radiomic features were identified as robust, meeting the criteria of a CCC greater than 0.99 for both inter-dose and inter-reconstruction comparisons, and a CV of less than 10% (Supplemental material).

## Discussion

So far, few studies have investigated the reproducibility of radiomic features derived from the advanced reconstruction algorithm of DLIR, despite its demonstrated potential for dose reduction, improved image quality, and enhanced pulmonary nodule visualization [14]. In this study, we evaluated the reproducibility of radiomic features extracted from DLIR images under different radiation doses and in comparisons with FBP and ASIR-V algorithms, stratified by nodule size and type. Between the standard-dose and low-dose, our findings demonstrated high reproducibility of DLIR-derived features, suggesting the suitability of DLIR-reconstructed images for radiomics analysis. For the texture features, DLIR-H showed superior inter-dose reproducibility (0.914 ± 0.119), compared to FBP (0.897 ± 0.127, *p* = 0.023). Under the low radiation dose, the reproducibility declined with increasing DLIR strength. Against ASIR-V50%, DLIR-L achieved higher reproducibility than FBP (0.950 ± 0.094 vs 0.943 ± 0.087, *p* = 0.001) and showed comparable performance to ASIR-V-50% against FBP (0.932 ± 0.118 vs 0.943 ± 0.087, *p* > 0.999). Part-solid nodules demonstrated the lowest variability (7.17 ± 13.48%) compared to solid nodules (8.24 ± 9.06%) and GGNs (13.24 ± 34.21%) (*p* < 0.001 for both). Variability decreased significantly with increasing nodule size (*p* < 0.001).

Our inter-dose analysis revealed that DLIR-H achieved superior or non-inferior reproducibility of radiomic features, particularly texture features, compared to FBP and ASIR-V-50%. This improvement may be attributed to the advanced noise-reduction capabilities of DLIR-H, as well as its enhanced texture preservation resulting from its training process. FBP provides limited noise suppression and produces images with substantial noise at low radiation doses [28], which may alter texture features and consequently impact radiomics reproducibility [18]. In contrast, the DLIR algorithm used in this study was trained on a large dataset of low-noise FBP images, which are widely considered the gold standard for image quality. The ability of DLIR to suppress noise while preserving fine image details contributes to more stable feature extraction. Our results are consistent with previous studies suggesting improved reproducibility with DLIR at low doses [17, 29]. These results suggest that radiomic models derived from DLIR-reconstructed low-dose CT images may achieve greater reliability and have enhanced potential for seamless translation between screening and diagnostic workflows.

Regarding inter-reconstruction reproducibility, our findings revealed a notable decrease in reproducibility with increasing DLIR strength when compared with the conventional reconstruction algorithms of FBP and ASIR-V50%. This may be attributed to the higher noise in FBP/ASIR-V images. Different denoising algorithms can introduce different noise textures and pixel-level variations across images [28, 30]. As DLIR strength increases, image quality diverges further from the high-noise FBP reference, consistent with the observed decline in reproducibility. Previous studies have similarly reported decreased robustness of radiomic features with stronger reconstruction settings for both ASIR-V and DLIR. [17, 31]. Although protocol heterogeneity is known to affect feature robustness, our findings highlight that understanding variations between DLIR and conventional reconstruction algorithms is essential for reliable clinical application of radiomic models, and that consistent reconstruction settings remain the most effective strategy to preserve model validity.

Texture features quantify the spatial distribution of voxel intensities, reflecting image heterogeneity [32, 33]. Consequently, divergences in texture-based features may indicate alterations across different spatial scales, potentially affecting radiologists’ clinical evaluation and the performance of radiomic models [34]. Texture features in our study demonstrated lower reproducibility than first-order and shape-based features across both dose levels and reconstruction methods. Therefore, differences in reproducibility among radiomic feature categories should be taken into account when developing multimodal predictive models. Reproducibility-based feature screening and prioritizing features with high stability across acquisition and reconstruction settings are recommended. In addition, given the development of reconstruction algorithms and application of radiomics, radiomic stability should be considered an important metric for evaluating the growing number of new deep learning-based reconstruction algorithms for low-dose CT, complementing traditional metrics such as the structural similarity index and peak signal-to-noise ratio.

Subgroup analysis revealed that radiomics reproducibility varied with nodule characteristics. Variability significantly decreased with increasing nodule size (*p* < 0.001). Consistently, Zhu et al reported that smaller nodules posed greater challenges to segmentation accuracy [35]. Increased segmentation variability may lead to instability and lower reproducibility of radiomic features. Prior research also indicated that while nodules over 1 cm were less affected, smaller nodules were more susceptible to acquisition parameters [36]. Regarding nodule type, part-solid nodules showed lower variability than solid nodules and pure GGNs (both *p* < 0.001). Pure GGNs, characterized by their low contrast and fuzzy borders, are more susceptible to noise, making segmentation and feature extraction more challenging. It has been consistently reported that pure GGNs have the lowest reproducibility [35]. However, Zheng et al found better reproducibility for GGNs than for solid nodules [29]. This discrepancy is likely explained by the smaller size of solid nodules in their cohort (5.54 ± 1.74 mm) compared with those in our study (17.55 ± 7.19 mm). Given our results regarding size and those of previous studies, nodule size appears to be a more dominant factor in feature stability than the internal density of the nodule itself. Differences in variability highlight the importance of considering nodule size and type when interpreting radiomic results in clinical applications.

Our study has some limitations. Firstly, as this was a single-center study using a single CT scanner, future multicenter studies with diverse vendors are warranted to validate the robustness of DLIR-based radiomic features. Secondly, DLIR-M and DLIR-L reconstructions were not available for the standard-dose CT scans, as only DLIR-H, FBP, and ASIR-V-50% images were acquired during the initial acquisition. This precludes a comprehensive evaluation of radiomic feature reproducibility across different DLIR strengths in standard-dose imaging, which warrants further investigation. Thirdly, our cohort included a wide spectrum of histological subtypes; therefore, future studies with larger, subtype-specific cohorts may further investigate the influence of histology on radiomic feature robustness.

In conclusion, our study provides strong evidence of the high reproducibility of radiomic features derived from DLIR-reconstructed CT images. Superior inter-dose reproducibility of DLIR-H indicates its promise for radiomic studies based on low-dose CT. However, caution is advised when selecting DLIR strength, as higher levels of DLIR may decrease reproducibility against ASIR-V or FBP. Inherent nodule characteristics, specifically size and type, significantly influence feature variability, indicating that future radiomics studies should account for these factors. A total of 21 original radiomic features were identified as robust, providing a stable candidate pool for future radiomics studies. These insights are crucial for optimizing CT acquisition and reconstruction protocols to ensure the robustness and clinical utility of radiomic biomarkers.

## Supplementary information

ELECTRONIC SUPPLEMENTARY MATERIAL

## Data Availability

The datasets used and analyzed during the current study are available from the corresponding author upon reasonable request.

## Abbreviation

ASIR-V-50%: Adaptive statistical iterative reconstruction-V at the 50% strength
ASIR-V: Adaptive statistical iterative reconstruction-V
CCC: Concordance correlation coefficient
CT: Computed tomography
CV: Coefficient of variation
DLIR-H: Deep learning image reconstruction at high strength
DLIR-L: Deep learning image reconstruction at low strength
DLIR-M: Deep learning image reconstruction at medium strength
DLIR: Deep learning image reconstruction
FBP: Filtered back projection
GGN: Ground-glass nodule
ROI: Region-of-interest
