# Supplementary material for: Impact of deep learning image reconstruction on CT radiomics reproducibility in patients with pulmonary nodules
Source: Insights Imaging. 2026 Oct 13;17:254. doi: 10.1186/s13244-026-02417-y (PMC13661886; doi:10.1186/s13244-026-02417-y)

Impact of Deep Learning Image Reconstruction on CT Radiomics Reproducibility in Patients with Pulmonary Nodules

ELECTRONIC SUPPLEMENTARY MATERIAL

1. Filters used in radiomic features extraction

For each ROI, a total of 2264 features were extracted from both the original and filtered images. A total of 24 different filtered image sets were generated by applying individual filters separately. These filters included Box Mean, Additive Gaussian Noise, Binomial Blur, Curvature Flow, Box-sigma, Normalize, Laplacian Sharpening, Discrete Gaussian, Mean, Speckle Noise, Recursive Gaussian, Shot noise, LoG (sigma value of 0.5, 1, 2, and 4), and Wavelet (LLL, LLH, LHL, LHH, HLL, HLH, HHL, and HHH).

2. Radiomic features

The extracted features were grouped into three categories: first-order features, shape-based features, and texture features. The details are as follows.

(1) First-order features (n=18): firstorder_10Percentile, firstorder_90Percentile, firstorder_Energy, firstorder_Entropy, firstorder_InterquartileRange, firstorder_Kurtosis, firstorder_Maximum, firstorder_MeanAbsoluteDeviation, firstorder_Mean, firstorder_Median, firstorder_Minimum, firstorder_Range, firstorder_RobustMeanAbsoluteDeviation, firstorder_RootMeanSquared, firstorder_Skewness, firstorder_TotalEnergy, firstorder_Uniformity, and firstorder_Variance.

(2) Shape-based features (n=14, only extracted from the original images): shape_Elongation, shape_Flatness, shape_LeastAxisLength, shape_MajorAxisLength, shape_Maximum2DDiameterColumn, shape_Maximum2DDiameterRow, shape_Maximum2DDiameterSlice, shape_Maximum3DDiameter, shape_MeshVolume, shape_MinorAxisLength, shape_Sphericity, shape_SurfaceArea, shape_SurfaceVolumeRatio, and shape_VoxelVolume.

(3) Texture features (n=72):

21 Gray Level Co-occurrence Matrix (GLCM) features including glcm_Autocorrelation, glcm_JointAverage, glcm_ClusterProminence, glcm_ClusterShade, glcm_ClusterTendency, glcm_Contrast, glcm_Correlation, glcm_DifferenceAverage, glcm_DifferenceEntropy, glcm_DifferenceVariance, glcm_JointEnergy, glcm_JointEntropy, glcm_InverseVariance, glcm_MaximumProbability, glcm_SumEntropy, glcm_Idn, glcm_Id, glcm_Idmn, glcm_Idm, glcm_Imc2, and glcm_Imc1.

16 Gray Level Run Length Matrix (GLRLM) features including glrlm_GrayLevelNonUniformity, glrlm_GrayLevelNonUniformityNormalized, glrlm_GrayLevelVariance, glrlm_HighGrayLevelRunEmphasis, glrlm_LongRunEmphasis, glrlm_LongRunHighGrayLevelEmphasis, glrlm_LongRunLowGrayLevelEmphasis, glrlm_LowGrayLevelRunEmphasis, glrlm_RunEntropy, glrlm_RunLengthNonUniformity, glrlm_RunLengthNonUniformityNormalized, glrlm_RunPercentage, glrlm_RunVariance, glrlm_ShortRunEmphasis, glrlm_ShortRunHighGrayLevelEmphasis, and glrlm_ShortRunLowGrayLevelEmphasis.

16 Gray Level Size Zone Matrix (GLSZM) features including glszm_GrayLevelNonUniformity, glszm_GrayLevelNonUniformityNormalized, glszm_GrayLevelVariance, glszm_HighGrayLevelZoneEmphasis,

glszm_LargeAreaEmphasis, glszm_LargeAreaHighGrayLevelEmphasis, glszm_LargeAreaLowGrayLevelEmphasis, glszm_LowGrayLevelZoneEmphasis, glszm_SizeZoneNonUniformity, glszm_SizeZoneNonUniformityNormalized, glszm_SmallAreaEmphasis, glszm_SmallAreaHighGrayLevelEmphasis, glszm_SmallAreaLowGrayLevelEmphasis, glszm_ZoneEntropy, glszm_ZonePercentage, and glszm_ZoneVariance.

14 Gray Level Dependence Matrix (GLDM) features including gldm_DependenceEntropy, gldm_DependenceNonUniformity, gldm_DependenceNonUniformityNormalized, gldm_DependenceVariance, gldm_GrayLevelNonUniformity, gldm_GrayLevelVariance, gldm_HighGrayLevelEmphasis, gldm_LargeDependenceEmphasis, gldm_LargeDependenceHighGrayLevelEmphasis, gldm_LargeDependenceLowGrayLevelEmphasis, gldm_LowGrayLevelEmphasis, gldm_SmallDependenceEmphasis, gldm_SmallDependenceHighGrayLevelEmphasis, and gldm_SmallDependenceLowGrayLevelEmphasis.

5 Neighboring Gray Tone Difference Matrix (NGTDM) features including ngtdm_Busyness, ngtdm_Coarseness, ngtdm_Complexity, ngtdm_Contrast, and ngtdm_Strength.

3. Power calculation

A post-hoc power analysis was performed using PASS 15 software (NCSS, Kaysville, Utah, USA) to validate the sample size. Based on a two-sided α of 0.05 and the observed Lin's Concordance Correlation Coefficient (CCC) values, the statistical power for all inter-dose and inter-reconstruction comparisons exceeded 0.999 (Table S1). This confirms that the current sample size of 94 patients is sufficient to ensure the reliability of our findings.

4. Robust features

A total of 21 original radiomic features were identified as robust, meeting the criteria of a concordance correlation coefficient greater than 0.99 for both inter-dose and inter-reconstruction comparisons, and a coefficient of variation of less than 10%. The robust features are listed below: firstorder_Energy, firstorder_TotalEnergy, firstorder_90Percentile, firstorder_RootMeanSquared, firstorder_Mean, firstorder_Median, firstorder_MeanAbsoluteDeviation, firstorder_RobustMeanAbsoluteDeviation, shape_MeshVolume, shape_VoxelVolume, shape_MinorAxisLength, shape_SurfaceArea, shape_LeastAxisLength, shape_MajorAxisLength, shape_Maximum2DDiameterColumn, shape_Maximum3DDiameter, shape_Maximum2DDiameterSlice, shape_Maximum2DDiameterRow, gldm_GrayLevelNonUniformity, glrlm_RunLengthNonUniformity, and glrlm_GrayLevelNonUniformity

Table S1. Power Calculation for All Inter-dose and Inter-reconstruction Comparisons

Comparisons		CCC	*P* value
Inter-dose	FBP	0.922	>0.999
	ASIR-V-50%	0.922	>0.999
	DLIR-H	0.934	>0.999
Inter-reconstruction	FBP vs. ASIR-V-50%	0.943	>0.999
	DLIR-H vs. FBP	0.909	>0.999
	DLIR-M vs. FBP	0.923	>0.999
	DLIR-L vs. FBP	0.932	>0.999
	DLIR-H vs. ASIR-V-50%	0.933	>0.999
	DLIR-M vs. ASIR-V-50%	0.938	>0.999
	DLIR-L vs. ASIR-V-50%	0.950	>0.999

CCC: concordance correlation coefficient

FBP: filtered back projection

ASIR-V-50%: adaptive statistical iterative reconstruction-V level 50%

DLIR-H: high-strength deep learning image reconstruction

DLIR-M: medium-strength deep learning image reconstruction

DLIR-L: low-strength deep learning image reconstruction

Table S2. Concordance Correlation Coefficients of Radimoic Features of Different Filters between Standard-dose and Low-dose CT

Filters	FBP	ASIR-V-50%	DLIR-H	*P* value
Original images	0.922±0.113	0.922±0.128	0.934±0.104	0.055
Box Mean	0.898±0.161	0.872±0.213*	0.878±0.192	0.035
Additive Gaussian Noise	0.894±0.146*	0.905±0.135	0.919±0.120	<0.001
Binomial Blur	0.906±0.151*	0.899±0.152*	0.880±0.188	<0.001
Curvature Flow	0.897±0.165*	0.906±0.153	0.911±0.145	<0.001
Box-sigma	0.897±0.161*	0.894±0.182*	0.921±0.163	<0.001
Normalize	0.850±0.140	0.859±0.162	0.798±0.205	0.007
Laplacian Sharpening	0.876±0.133	0.857±0.187	0.881±0.158	0.034
Discrete Gaussian	0.912±0.152	0.914±0.144	0.915±0.126	0.261
Mean	0.912±0.129*	0.895±0.164*	0.876±0.191	<0.001
Speckle Noise	0.935±0.117*	0.930±0.117	0.931±0.109	<0.001
Recursive Gaussian	0.909±0.151*	0.911±0.164	0.909±0.147	0.001
Shot noise	0.902±0.129	0.911±0.133*	0.899±0.138	<0.001
LoG	0.853±0.154*	0.835±0.186	0.838±0.178	<0.001
Wavelet	0.727±0.237	0.741±0.227*	0.745±0.211	<0.001

FBP: filtered back projection

ASIR-V-50%: adaptive statistical iterative reconstruction-V level 50%

DLIR-H: high-strength deep learning image reconstruction

*: $P<0.05$, compared with concordance correlation coefficient of DLIR-H

Table S3. Concordance Correlation Coefficients of Radimoic Features of Different Filters among Low-dose CT Reconstructed with Different Algorithm

Filters	FBP vs. ASIR-V-50%	DLIR vs. FBP			DLIR vs. ASIR-V-50%			*P* value
		DLIR-H	DLIR-M	DLIR-L	DLIR-H	DLIR-M	DLIR-L	
Original images	0.943±0.087	0.909±0.138*	0.923±0.126*	0.932±0.118	0.933±0.116	0.938±0.116	0.950±0.094*	<0.001
Box Mean	0.916±0.170	0.900±0.168*	0.902±0.164*	0.920±0.151	0.921±0.144	0.926±0.139	0.932±0.130	<0.001
Additive Gaussian Noise	0.895±0.138	0.902±0.142	0.930±0.114*	0.939±0.105*	0.927±0.108*	0.909±0.131*	0.904±0.123	<0.001
Binomial Blur	0.926±0.128	0.891±0.187*	0.903±0.173*	0.902±0.182*	0.920±0.146*	0.913±0.164	0.907±0.169	<0.001
Curvature Flow	0.938±0.099	0.917±0.131*	0.923±0.122	0.927±0.135	0.935±0.095	0.927±0.119	0.916±0.123*	<0.001
Box-sigma	0.942±0.100	0.901±0.162*	0.907±0.157*	0.931±0.130	0.912±0.166*	0.918±0.150*	0.932±0.129	<0.001
Normalize	0.858±0.168	0.833±0.150*	0.876±0.128*	0.846±0.166	0.833±0.178*	0.876±0.143	0.910±0.116*	<0.001
Laplacian Sharpening	0.913±0.126	0.868±0.166*	0.897±0.129	0.907±0.157	0.876±0.142*	0.883±0.144*	0.900±0.154	<0.001
Discrete Gaussian	0.941±0.083	0.928±0.124	0.935±0.104	0.935±0.104*	0.948±0.103	0.951±0.081*	0.937±0.092	<0.001
Mean	0.921±0.140	0.892±0.174*	0.906±0.155*	0.916±0.154	0.917±0.152	0.917±0.163	0.928±0.149	<0.001
Speckle Noise	0.947±0.095	0.930±0.142	0.937±0.120	0.946±0.100	0.937±0.114	0.923±0.141*	0.948±0.089	<0.001
Recursive Gaussian	0.941±0.088	0.915±0.140	0.907±0.157	0.916±0.157*	0.917±0.123*	0.904±0.148*	0.904±0.150*	<0.001
Shot noise	0.935±0.107	0.908±0.132*	0.916±0.132*	0.926±0.118	0.921±0.118*	0.924±0.114*	0.933±0.110	<0.001
LoG	0.900±0.125	0.832±0.167*	0.854±0.153*	0.868±0.147*	0.876±0.149*	0.888±0.144*	0.899±0.134	<0.001
Wavelet	0.830±0.170	0.711±0.224*	0.751±0.205*	0.770±0.202*	0.773±0.196*	0.807±0.189*	0.828±0.186*	<0.001

FBP: filtered back projection

ASIR-V-50%: adaptive statistical iterative reconstruction-V level 50%

DLIR-H: high-strength deep learning image reconstruction

DLIR-M: medium-strength deep learning image reconstruction

DLIR-L: low-strength deep learning image reconstruction

*: $P<0.05$, compared with concordance correlation coefficient of FBP vs. ASIR-V-50%

Table S4. Coefficient of Variations of Radimoic Features of Different Filters

Filters	Overall	Ground glass nodule	Part-solid nodule	Solid nodule	<10mm	10–20mm	20–30mm
Original images	9.87±20.32%	13.24±34.21%	7.17±13.48%	8.24±9.06%	14.18±39.23%	8.46±14.30%	5.16±6.47%
Box Mean	10.68±11.46%	15.05±15.33%	7.86±9.68%	6.90±12.30%	15.59±16.31%	8.94±10.80%	5.56±7.72%
Additive Gaussian Noise	13.11±19.25%	14.08±30.22%	12.16±14.58%	13.02±12.01%	15.05±34.04%	9.73±14.42%	14.56±13.47%
Binomial Blur	10.97±14.65%	16.23±23.82%	7.46±9.92%	6.71±9.38%	15.83±25.95%	9.72±11.51%	5.06±6.84%
Curvature Flow	16.58±50.96%	14.06±18.12%	10.98±12.12%	36.39±255.07%	15.42±24.30%	19.86±101.53%	11.15±13.43%
Box-sigma	11.80±11.70%	13.49±13.78%	10.94±11.43%	9.80±14.04%	8.54±9.92%	15.10±16.62%	9.23±8.90%
Normalize	13.20±23.04%	18.72±37.94%	10.27±16.77%	6.88±7.68%	19.90±43.47%	10.40±16.17%	7.06±9.49%
Laplacian Sharpening	12.60±12.23%	13.40±13.79%	11.52±13.46%	13.25±9.18%	13.72±15.00%	9.91±12.22%	14.08±10.44%
Discrete Gaussian	12.12±14.21%	14.42±14.03%	11.59±16.82%	7.78±11.95%	17.11±17.85%	8.55±14.24%	9.76±14.07%
Mean	10.29±11.23%	14.67±15.83%	7.40±9.00%	6.66±10.82%	14.87±17.10%	8.60±9.08%	5.59±7.85%
Speckle Noise	8.40±6.72%	9.75±8.29%	8.45±6.72%	5.02±4.81%	9.16±7.42%	7.47±6.79%	7.87±6.72%
Recursive Gaussian	11.91±12.36%	13.39±12.28%	10.91±13.94%	10.74±11.91%	13.54±15.36%	9.54±9.37%	12.22±15.07%
Shot noise	9.40±9.57%	11.32±10.63%	7.63±8.61%	9.06±10.70%	11.91±10.62%	8.24±10.12%	6.81±8.23%
LoG	16.99±29.02%	22.95±56.66%	14.20±20.73%	9.26±12.15%	20.92±33.17%	17.05±49.00%	9.33±11.37%
Wavelet	21.24±59.84%	25.95±93.18%	18.82±47.44%	16.63±46.58%	24.85±103.54%	19.85±56.40%	16.58±34.66%

Figure S1 and Legend

Figure S1. Images of a 67-year-old female patient with a solid nodule in the right lower lobe of the lung, pathologically confirmed as invasive adenocarcinoma. Images reconstructed with high-strength deep learning image reconstruction (DLIR-H) show lower image noise compared with adaptive statistical iterative reconstruction V at 50% strength (ASIR-V-50%) and filtered back projection (FBP). The objective noise measurement (A). A fixed-size circular region-of-interest (ROI) was placed in the trachea, and objective noise was quantified as the standard deviation of Hounsfield units within the ROI. Low-dose CT images were reconstructed using DLIR at low strength (B), DLIR at medium strength (C), DLIR-H (D), ASIR-V-50% (E), and FBP (F). Standard-dose CT images were reconstructed using DLIR-H (G), ASIR-V-50% (H), and FBP (I). Boxes with magnified illustrations of the nodules have been added to improve visibility.

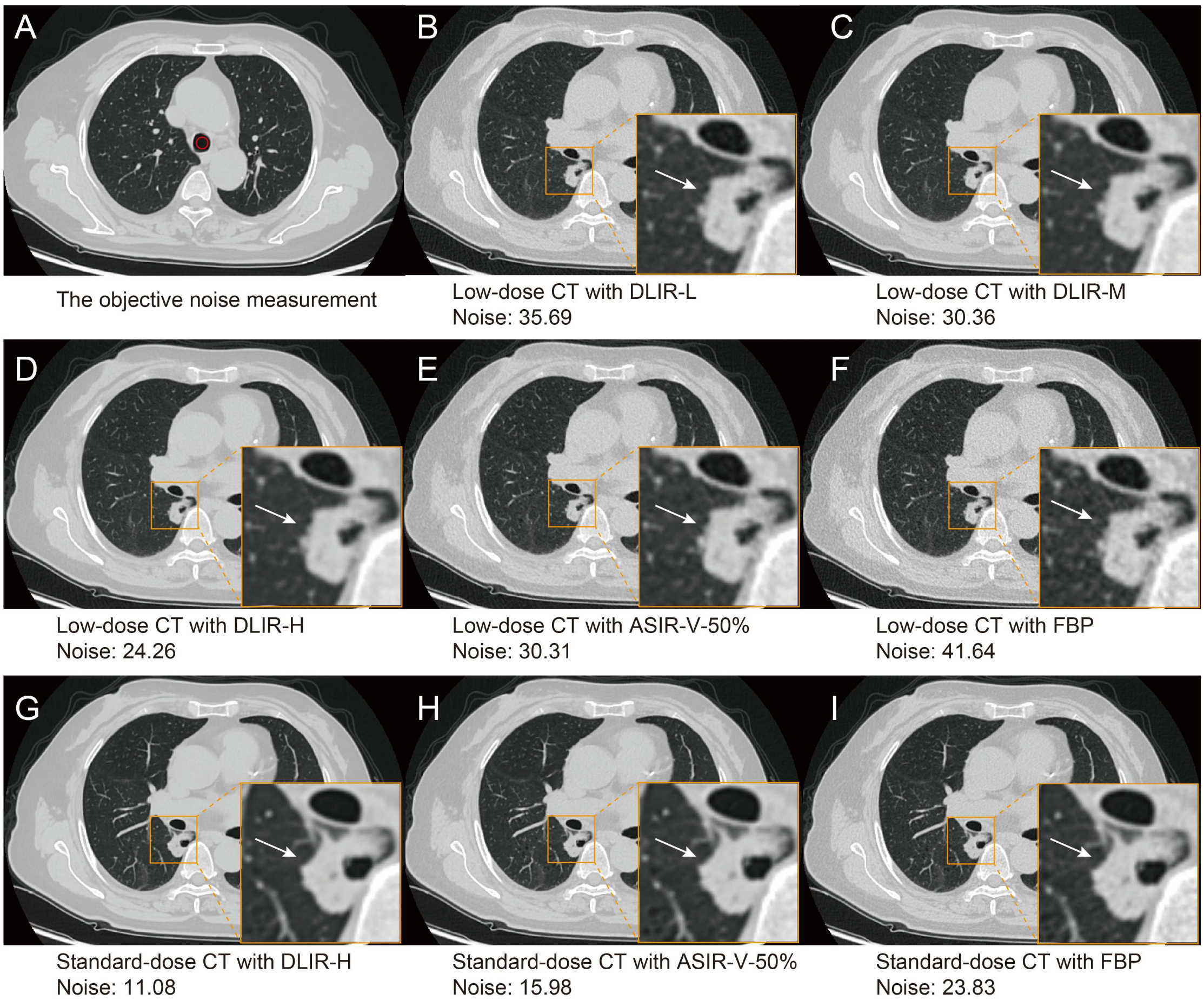

Supplement: Supplementary file 1 [file 13244_2026_2417_MOESM1_ESM.pdf]
